# Supplementary material for: An alpaca nanobody neutralizes SARS-CoV-2 by blocking receptor interaction
Source: Nat Commun. 2020 Sep 4;11:4420. doi: 10.1038/s41467-020-18174-5 (PMC7473855; doi:10.1038/s41467-020-18174-5)
Supplement: Supplementary file 1 — Supplementary Information [file 41467_2020_18174_MOESM1_ESM.pdf]

Supplemental Information for

An alpaca nanobody neutralizes SARS-CoV-2 by blocking receptor interaction

by

Leo Hanke, Laura Vidakovics Perez, Daniel J. Sheward, Hrishikesh Das, Tim Schulte, Ainhoa Moliner-Morro, Martin Corcoran, Adnane Achour, Gunilla B. Karlsson Hedestam, B. Martin Hällberg, Ben Murrell and Gerald M. McInerney

Supplemental Table 1 Cryo-EM data collection, refinement and validation statistics

	#1 Spike-Ty1<br>(EMDB-11526)<br>(PDB 6ZXN)
**Data collection and processing**	
Magnification	165,000x
Voltage (kV)	300 kV
Electron exposure (e–/Å^2)	38
Defocus range (μm).	0.5 – 2.0 microns
Pixel size (Å).	0.51 Å
Symmetry imposed	C1
Initial particle images (no.)	573,036
Final particle images (no.)	210,832
Map resolution (Å)<br>FSC threshold	0.143 FSC
Map resolution range (Å)	2.9 Å
**Refinement**	
Initial model used (PDB code)	6VSB, 5JMR, 6LZG
Model resolution (Å)<br>FSC threshold	2.9 Å<br>0.143
Model resolution range (Å)	2.9 – 3.6
Map sharpening *B* factor (Å^2)	-67
Model composition<br>Non-hydrogen atoms<br>Protein residues<br>Ligands	 26297<br>3261<br>67 (NAG)
B factors (Å^2)<br>Protein<br>Ligand	 137<br>129
R.m.s. deviations<br>Bond lengths (Å)<br>Bond angles (°)	 0.012<br>1.459
Validation<br>MolProbity score<br>Clashscore<br>Poor rotamers (%)	 2.87<br>38.66<br>0.10
Ramachandran plot<br>Favored (%)<br>Allowed (%)<br>Disallowed (%)	 95.53<br>4.47<br>0.00

Supplemental Table 2 List of primers used in the study.

Description	Name	Sequences
Gene specific primers for cDNA synthesis	AL.CH2	ATGGAGAGGACGTCCTTGGGT
	AL.CH2.2	TTCGGGGGGAAGAYRAAGAC
Primers for library amplification and cloning into phagemid plasmid	AIVHH-F1	CTTGCGGCCGCTCAGKTGCAGCTCGTGGAGWCNGGNGG
	AIVHH-shR1	GATCGGCGCGCCGAGGGGTCTTCGCTGTGGTGCG
	AIVHH-lhR1	GATCGGCGCGCCGGTTGTGGTTTTGGTGTCTTGGG
Next generation sequencing primers	NB-NGS-FW	CACTCTTTCCCTACACGACGCTCTTCCGATCTCTCGCGGCCCAGCCGGCCATGG
	NB-NGS-RV	GGAGTTCAGACGTGTGCTCTTCCGATCTACCGGCGCACCACTAGTGCA
Primers for cloning nanobodies into pHEN plasmid using Gibson assembly	nb-FW	CGCGGCCCAGCCGGCCATGGCCCAGGTGCAGCTCGTGG
	nb-con-Rev	AGTCCTCCTGAGGAGACGGTGACCTGGGTCCCCTGGCC

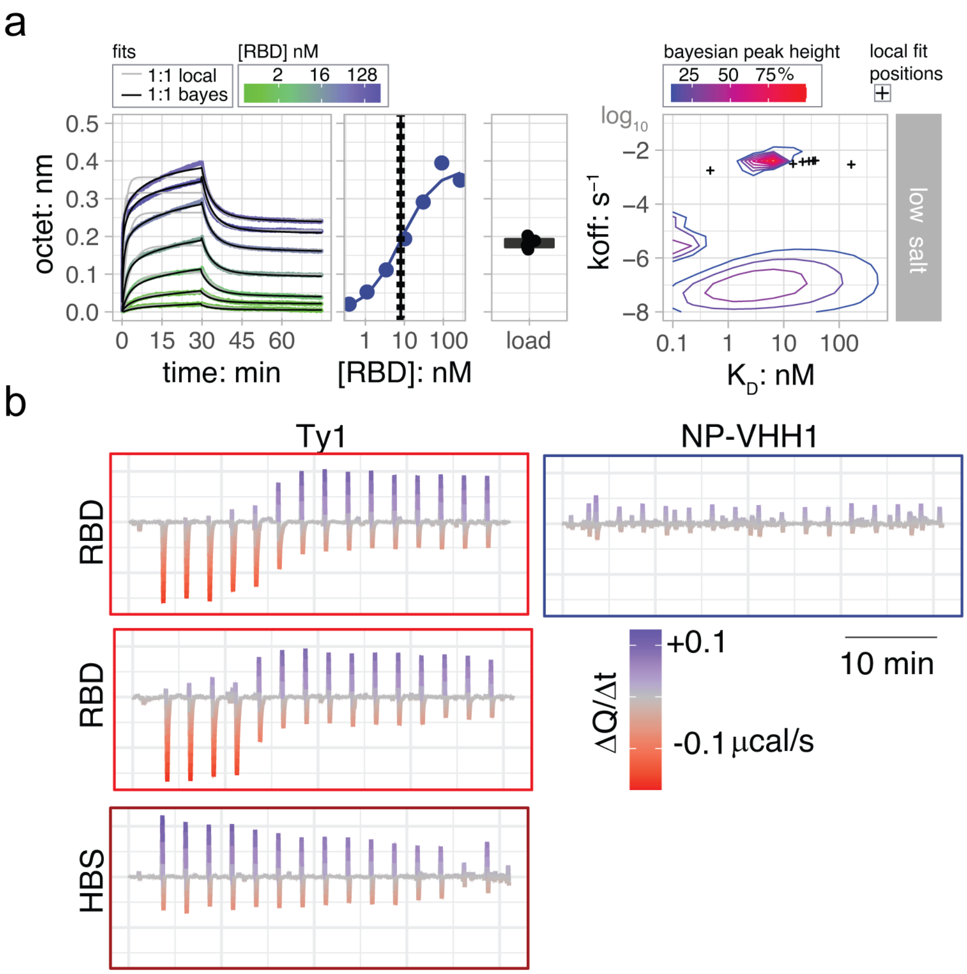

Supplemental Figure 1 a, related to Fig 3b, plots for RBD/Ty1 titrations at low salt condition with same legends and scales. **b**, related to Fig 3c, Same data plotted on larger scale to highlight the Ty1 into buffer dilution spikes.

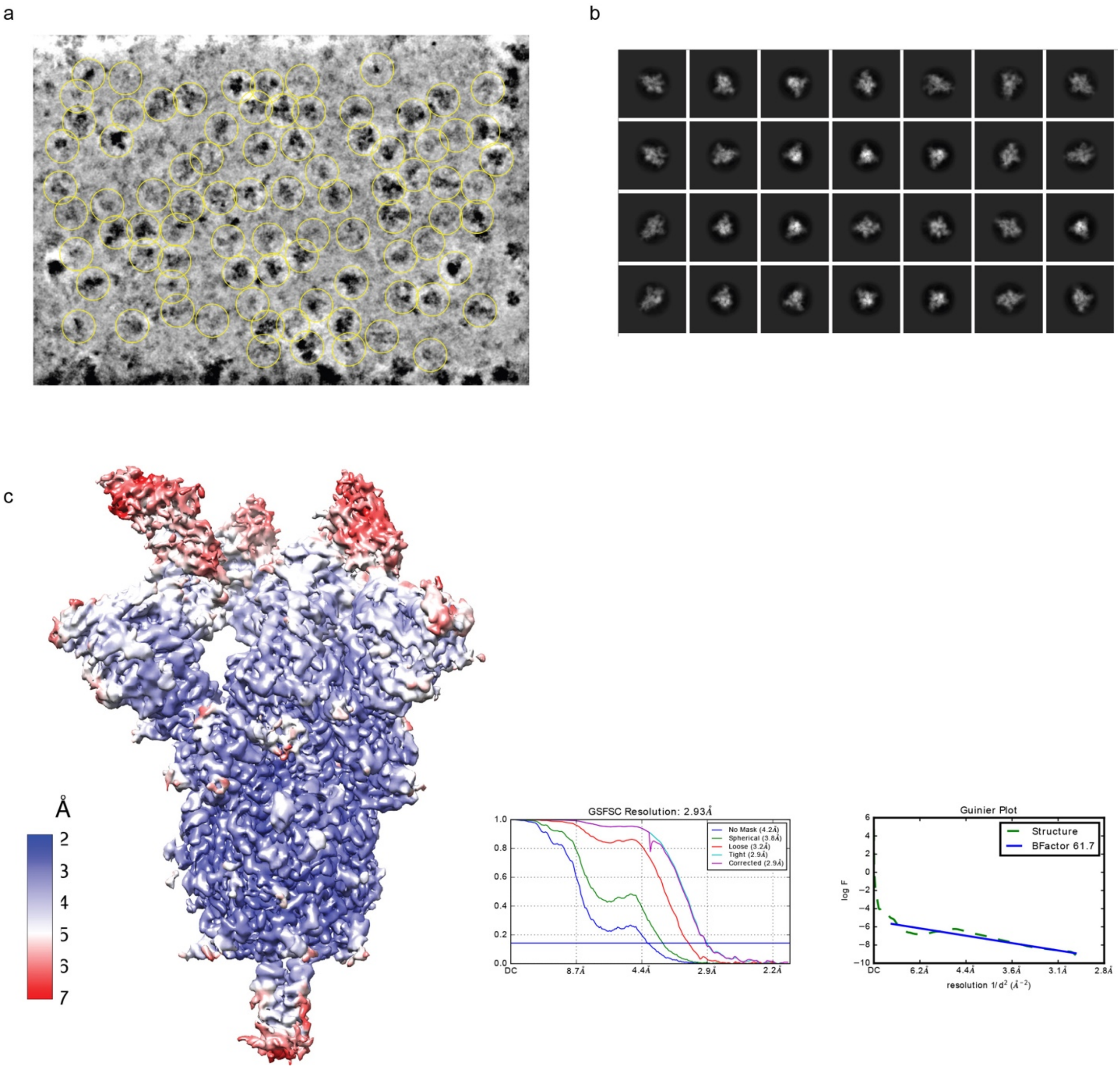

Supplemental Figure 2 Cryo-EM processing

a, Representative micrographs. For clarity, the micrographs has been denoised using Warp. Particle autopicked by Warp are denoted with a yellow ring with 200 Å diameter in the sample plane. **b**, Representative 2D classes from CryoSPARC. **c**, Local reconstruction resolution mapped to a locally sharpened map. **d**, FSC curve and Guinier plot as calculated by CryoSPARC.
