# Supplementary material for: An alpaca nanobody neutralizes SARS-CoV-2 by blocking receptor interaction
Source: Nat Commun. 2020 Sep 4;11:4420. doi: 10.1038/s41467-020-18174-5 (PMC7473855; doi:10.1038/s41467-020-18174-5)
Supplement: Supplementary file 3 — Reporting Summary [file 41467_2020_18174_MOESM3_ESM.pdf]

nature research

Corresponding author(s): B. Martin Hällberg
Ben Murrell
Gerald M. McInerney

Last updated by author(s): Jul 25, 2020

# Reporting Summary

Nature Research wishes to improve the reproducibility of the work that we publish. This form provides structure for consistency and transparency in reporting. For further information on Nature Research policies, see our Editorial Policies and the Editorial Policy Checklist.

## Statistics

For all statistical analyses, confirm that the following items are present in the figure legend, table legend, main text, or Methods section.

| n/a | Confirmed | |
|---|---|---|
| x | | The exact sample size (*n*) for each experimental group/condition, given as a discrete number and unit of measurement |
| x | | A statement on whether measurements were taken from distinct samples or whether the same sample was measured repeatedly |
| x | | The statistical test(s) used AND whether they are one- or two-sided<br>*Only common tests should be described solely by name; describe more complex techniques in the Methods section.* |
| x | | A description of all covariates tested |
| x | | A description of any assumptions or corrections, such as tests of normality and adjustment for multiple comparisons |
| x | | A full description of the statistical parameters including central tendency (e.g. means) or other basic estimates (e.g. regression coefficient) AND variation (e.g. standard deviation) or associated estimates of uncertainty (e.g. confidence intervals) |
| x | | For null hypothesis testing, the test statistic (e.g. $F$, $t$, $r$) with confidence intervals, effect sizes, degrees of freedom and $P$ value noted<br>*Give P values as exact values whenever suitable.* |
| | x | For Bayesian analysis, information on the choice of priors and Markov chain Monte Carlo settings |
| x | | For hierarchical and complex designs, identification of the appropriate level for tests and full reporting of outcomes |
| x | | Estimates of effect sizes (e.g. Cohen's $d$, Pearson's $r$), indicating how they were calculated |

*Our web collection on statistics for biologists contains articles on many of the points above.*

## Software and code

Policy information about availability of computer code

| | |
|---|---|
| Data collection | *Provide a description of all commercial, open source and custom code used to collect the data in this study, specifying the version used OR state that no software was used.* |
| Data analysis | Data analysis: Next generation sequencing data was processed in the Julia language (version 1.1.1), using the NextGenSeqUtils.jl package (version 1.0). Jupyter notebooks to reproduce the NGS data processing are available at: https://github.com/MurrellGroup/Ty1 |

For manuscripts utilizing custom algorithms or software that are central to the research but not yet described in published literature, software must be made available to editors and reviewers. We strongly encourage code deposition in a community repository (e.g. GitHub). See the Nature Research guidelines for submitting code & software for further information.

## Data

Policy information about availability of data

All manuscripts must include a data availability statement. This statement should provide the following information, where applicable:

- Accession codes, unique identifiers, or web links for publicly available datasets
- A list of figures that have associated raw data
- A description of any restrictions on data availability

The sequence of Ty1 was deposited in the NCBI GenBank sequence data base and is available under the accession code MT784731.
BLI and ITC data is available here https://github.com/derpaule/Ty1_octet_itc and here doi:10.5061/dryad.gb5mkkwmz. Next generation sequencing data is deposited at the SRA, under BioProject ID PRJNA638614. Jupyter notebooks to reproduce the NGS data processing are available at: ttps://github.com/MurrellGroup/Ty1. Other source data are provided with this paper.

# Field-specific reporting

Please select the one below that is the best fit for your research. If you are not sure, read the appropriate sections before making your selection.

☒ Life sciences ☐ Behavioural & social sciences ☐ Ecological, evolutionary & environmental sciences

For a reference copy of the document with all sections, see nature.com/documents/nr-reporting-summary-flat.pdf

# Life sciences study design

All studies must disclose on these points even when the disclosure is negative.

| | |
|---|---|
| Sample size | No sample-size calculation was performed as it is non relevant for this study. |
| Data exclusions | No data was excluded of any analysis performed |
| Replication | Replicated experiments are indicated in the figure legends |
| Randomization | This study focuses on functional and structural characterization of a novel nanobody, and randomization is not relevant. |
| Blinding | This study focuses on functional and structural characterization of a novel nanobody, and blinding is not relevant. |

# Reporting for specific materials, systems and methods

We require information from authors about some types of materials, experimental systems and methods used in many studies. Here, indicate whether each material, system or method listed is relevant to your study. If you are not sure if a list item applies to your research, read the appropriate section before selecting a response.

### Materials & experimental systems

| n/a | Involved in the study | |
|---|---|---|
| ☐ | ☒ | Antibodies |
| ☐ | ☒ | Eukaryotic cell lines |
| ☒ | ☐ | Palaeontology and archaeology |
| ☐ | ☒ | Animals and other organisms |
| ☒ | ☐ | Human research participants |
| ☒ | ☐ | Clinical data |
| ☒ | ☐ | Dual use research of concern |

### Methods

| n/a | Involved in the study | |
|---|---|---|
| ☒ | ☐ | ChIP-seq |
| ☐ | ☒ | Flow cytometry |
| ☒ | ☐ | MRI-based neuroimaging |

## Antibodies

| | |
|---|---|
| Antibodies used | anti ds-RNA mAb J2, SCICONS, Product No. 10010500, Batch No. J2-1915<br>Donkey anti-Mouse IgG (H+L) Alexa Fluor 488, Invitrogen, Cat# A-21202, Lot 2147618<br>E Tag Antibody, Bethyl laboratories, Cat# A190-133P |
| Validation | ds-RNA antibody was validated by titration using virus infected and un-infected cells. E tag antibody was validated by ELISA. |

## Eukaryotic cell lines

Policy information about cell lines

| | |
|---|---|
| Cell line source(s) | Cell lines were purchased from ATCC: HEK293T (ATCC-CRL-3216), Vero E6 (ATCC CRL-1586) or Thermo Fischer Scientific: FreeStyle 293-F |
| Authentication | HEK293T: authentication was not performed. Stock of cells was recently purchased from ATCC; Vero E6: authentication was not performed. This cell line does not belong to the list of frequently misidentified cell lines. |
| Mycoplasma contamination | All cell lines used for experiments were negative for Mycoplasma as determined by PCR. |
| Commonly misidentified lines (See ICLAC register) | *Name any commonly misidentified cell lines used in the study and provide a rationale for their use.* |

## Animals and other organisms

Policy information about studies involving animals; ARRIVE guidelines recommended for reporting animal research

| | |
|---|---|
| Laboratory animals | New world camelid, Alpaca, male, 9.4 years |
| Wild animals | This study did not involve wild animals |
| Field-collected samples | This study did not involve samples collected in the field. |
| Ethics oversight | All work involving animal immunizations at PreClinics GMBH complies with the relevant ethical regulations for animal testing and research. |

Note that full information on the approval of the study protocol must also be provided in the manuscript.

## Flow Cytometry

### Plots

Confirm that:

- [x] The axis labels state the marker and fluorochrome used (e.g. CD4-FITC).
- [x] The axis scales are clearly visible. Include numbers along axes only for bottom left plot of group (a 'group' is an analysis of identical markers).
- [ ] All plots are contour plots with outliers or pseudocolor plots.
- [ ] A numerical value for number of cells or percentage (with statistics) is provided.

### Methodology

| | |
|---|---|
| Sample preparation | Cells were trypsinized, and fixed in 4% PFA, and stained with Ty1-AS635P under permeabilizing conditions (0.1% saponin) or with RBD-AS635P under non-permeabilizing conditions. |
| Instrument | BD FACSCelesta |
| Software | Flow cytometry data was collected with BD FACS Diva software and analyzed with the FlowJo Software package |
| Cell population abundance | No sorting was performed with the flow cytometer |
| Gating strategy | For gating forward scatter vs side scatter was used to separate cell from debris, resulting events were displayed as a histogram |

- [ ] Tick this box to confirm that a figure exemplifying the gating strategy is provided in the Supplementary Information.
